# Recombinant rabies virus expressing dog GM-CSF is an efficacious oral rabies vaccine for dogs

**DOI:** 10.18632/oncotarget.5904

**Published:** 2015-09-30

**Authors:** Ming Zhou, Lei Wang, Songqin Zhou, Zhao Wang, Juncheng Ruan, Lijun Tang, Ziming Jia, Min Cui, Ling Zhao, Zhen F. Fu

**Affiliations:** ^1^ State Key Laboratory of Agricultural Microbiology, College of Veterinary Medicine, Huazhong Agricultural University, Wuhan, China; ^2^ Department of Pathology, University of Georgia, Athens, GA, USA; ^3^ Hubei Provincial Key Laboratory for Applied Toxicology, Hubei Provincial Academy of Preventive Medicine, Wuhan, China

**Keywords:** rabies, recombinant rabies virus, GM-CSF, oral vaccine, dog, Immunology and Microbiology Section, Immune response, Immunity

## Abstract

Developing efficacious oral rabies vaccines is an important step to increase immunization coverage for stray dogs, which are not accessible for parenteral vaccination. Our previous studies have demonstrated that recombinant rabies virus (RABV) expressing cytokines/chemokines induces robust protective immune responses after oral immunization in mice by recruiting and activating dendritic cells (DCs) and B cells. To develop an effective oral rabies vaccine for dogs, a recombinant attenuated RABV expressing dog GM-CSF, designated as LBNSE-dGM-CSF was constructed and used for oral vaccination in a dog model. Significantly more DCs or B cells were activated in the peripheral blood of dogs vaccinated orally with LBNSE-dGM-CSF than those vaccinated with the parent virus LBNSE, particularly at 3 days post immunization (dpi). As a result, significantly higher levels of virus neutralizing antibodies (VNAs) were detected in dogs immunized with LBNSE-dGM-CSF than with the parent virus. All the immunized dogs were protected against a lethal challenge with 4500 MICLD_50_ of wild-type RABV SXTYD01. LBNSE-dGM-CSF was found to replicate mainly in the tonsils after oral vaccination as detected by nested RT-PCR and immunohistochemistry. Taken together, our results indicate that LBNSE-dGM-CSF could be a promising oral rabies vaccine candidate for dogs.

## INTRODUCTION

Rabies is caused by the rabies virus (RABV) and is one of the oldest zoonoses in history. Today, it remains a public health threat causing more than 55,000 human deaths per year worldwide, most of which occurs in the developing countries of Asia and Africa [1]. In these places, infected dog bites are the major reason for the high incidence of human rabies, therefore, control of canine rabies is the most cost-effective approach to eliminate human rabies [2, 3]. It has been demonstrated that vaccination coverage of 70% of the canine population can efficiently reduce virus transmission and thus prevent human rabies [4, 5]. It was estimated that about 75% of dogs worldwide are free to roam [6]. In India, the highest proportion of ownerless dogs was reported in urban India, where the proportion of stray dogs (might include dogs that owned but were allowed to roam freely) to pet dog was 2:1[7, 8]; dogs were responsible for 96.2% of human rabies deaths, and the majority (75.3%) of these was stray dogs [9]. In China, more human rabies cases were associated with stray dogs rather than domesticated dogs [10] and the rabies vaccination coverage of stray dogs is almost zero [11]. In Bangkok, approximately 17% of dogs were considered to be ownerless [12]. The parenteral vaccination of these stray or owned but uncontrolled dogs is always difficult, laborious and not cost effective. Therefore, developing an efficient rabies vaccine for free-roaming dogs is crucial for rabies control in these countries.

Oral vaccination has been shown to be a practical way to control rabies for wildlife [13-17]. Currently, two oral rabies vaccines, SAG-2 and VR-G were recommended by WHO for dog vaccination [2]. VR-G is a recombinant vaccinia virus expressing RABV glycoprotein (G). [18] and has been successfully used for control of fox rabies in Europe and for coyote and raccoon rabies control in the United States [14, 15, 19-21]. However, it has been reported that two humans induced intensive skin inflammation and systemic vaccinia infection after VR-G exposure [22, 23], which could pose a safety issue for humans if used in dogs since humans contact closely with dogs. SAG-2 is an attenuated RABV derived from SAD-Bern strain (B 19) with two nucleotide mutations at its glycoprotein codon 333 [24, 25]. It has been used as an oral rabies vaccine in many animal species [26-29] and registered for canine rabies control in India [30]. However, the level of virus neutralizing antibodies (VNA) titers induced by SAG-2 is generally low in dogs after oral vaccination and not all vaccinated dogs develop a detectable VNA titer [26, 30, 31], which makes it difficult to determine the effectiveness of the vaccination. Another recombinant RABV, SPBNGAS-GAS, which expresses two copies of the G has also been used for oral immunization in dogs. Although this recombinant RABV was demonstrated to be very effective in protecting immunized dogs from lethal challenge, still one of six immunized dogs did not seroconvert by 14 dpi and the VNA titer was low [32]. Moreover, many other recombinant vectors have been developed as oral rabies vaccines for dogs, such as adenoviruses [33-35], parapoxvirus [36], and pseudorabies virus [37], nevertheless, VNA responses were generally low in dogs. Therefore, more efficacious oral vaccines are urgently needed for canine rabies control.

Our previous studies have shown that recombinant attenuated RABV expressing chemokines or cytokines enhance the immunogenicity by recruiting and activating dendritic cells (DCs) and B cells [38-41]. One of these viruses, RABV expressing murine granulocyte-macrophage colony-stimulating factor (GM-CSF), has been tested for its oral immunogenicity in a mouse model. It was shown that this recombinant virus stimulated higher immune responses and provided better protection in mice after oral immunization than the parent virus [38]. In the present study, a recombinant attenuated RABV expressing dog GM-CSF was constructed based on the parent virus LBNSE. The LBNSE is an attenuated RABV containing two mutations at amino acid 194 and 333 of its G protein, and the RABV with these two mutations has been demonstrated to be an avirulent phenotype in previous study [42]. It was found that the recombinant attenuated RABV expressing dog GM-CSF could activate more DCs and B cells in the peripheral blood and to induce significantly higher VNA titers after oral immunization than the parent virus in dogs.

## RESULTS

### Construction and characterization of recombinant attenuated RABV expressing dog GM-CSF (LBNSE-dGM-CSF)

**Figure 1:**
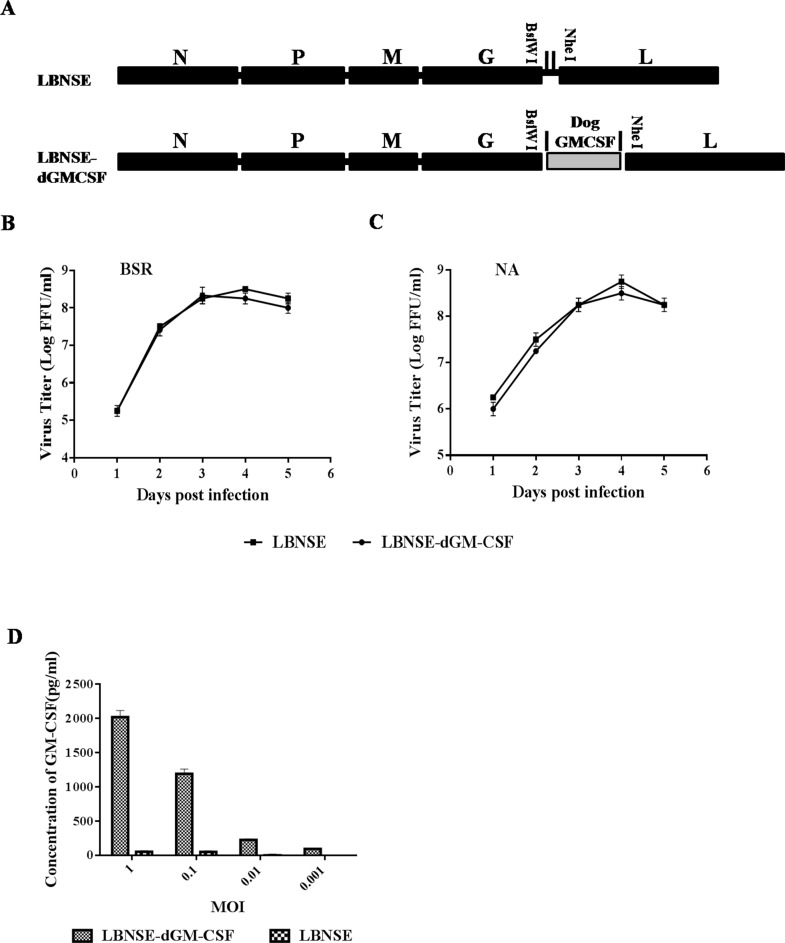
Construction and *in vitro* characterization of rRABV LBNSE-dGM-CSF **A.** Schematic diagram for the construction of recombinant LBNSE and LBNSE-dGM-CSF. The pLBNSE vector was derived from SAD-B19 with the deletion of the long non-coding region between RABV G and L genes and the insertion of BsiWI and NheI sites as described previously [39]. Dog GM-CSF gene was cloned and inserted into the RABV genome in the place of the deleted long non-coding region, the recombinant RABVs were rescued following the method described in Method section. **B.** and **C.** The growth curves of the recombinant RABVs determined on BSR cells and NA cells, respectively. Briefly, BSR or NA cells were infected with either LBNSE or LBNSE-dGM-CSF at a multiplicity of infection (MOI) of 0.01. The culture supernatants were collected at 1, 2, 3, 4 and 5 dpi, and viral titers determined. All the titrations were carried out in quadruplicate, and each value was expressed as the mean ± SEM from three independent experiments. **D.** The expression level of dog GM-CSF was determined by a commercial ELISA kit. Briefly, NA cells were infected with LBNSE-dGM-CSF or LBNSE (MOI=1, 0.1, 0.01, or 0.001) for 24 hrs, and the culture supernatants were harvested for measurement of dog GM-CSF, each value was expressed as mean ± SEM from three independent experiments.

Although recombinant RABV expressing murine GM-CSF has been constructed and shown to stimulate protective immunity after oral immunization in mice [38], dog GM-CSF was cloned into RABV as shown in Figure 1A to overcome the possible species specificity [43]. The recombinant RABV expressing dog GM-CSF is designated as LBNSE-dGM-CSF and was rescued using the procedures described previously [38]. The growth curve of LBNSE-dGM-CSF was determined on BSR or NA cells and compared with that of the parent virus LBNSE. As shown in Figure 1B and 1C, the growth curve of LBNSE-dGM-CSF is similar to that of LBNSE, indicating that the insertion of dog GM-CSF gene did not affect virus replication. In addition, the expression of GM-CSF was measured by ELISA. As shown in Figure 1D, dog GM-CSF is expressed in a dose-dependent manner in NA cells infected with LBNSE-dGM-CSF.

### Safety and viral replication in the oral cavity after vaccination in dogs

**Figure 2:**
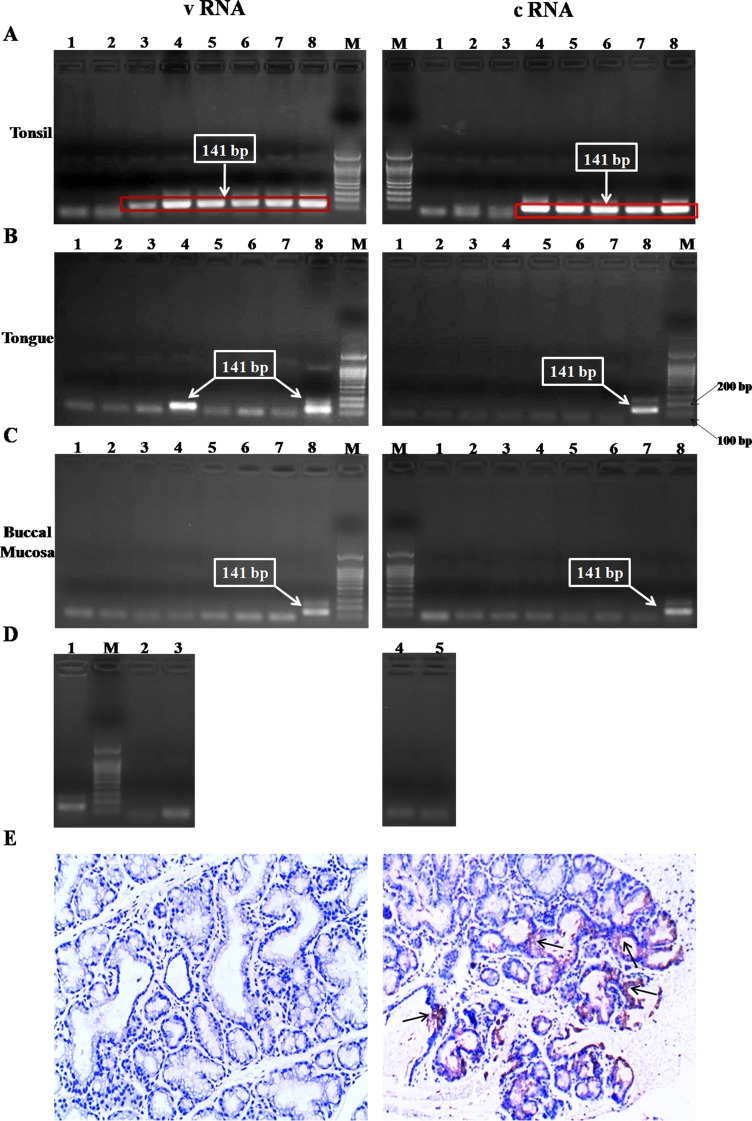
Detection of viral replication in the oral cavity after oral immunization by nested RT-PCR and IHC Dogs were orally sham-immunized or immunized with LBNSE-dGM-CSF, and samples/biopsies of tonsils, tongues, and buccal mucosa were collected at 24, 48, 72, and 96 hrs post immunization (hpi). Viral RNA was detected by nested RT-PCR **A.**, **B.**, **C.**, and **D.** is the internal reagents control for the nested RT-PCR. For **A.**, **B.**, and **C.**, the left panels depict the results for vRNA and the right panels are the results for cRNA detection; lane M represents DNA ladder marker; lanes 1 and 2 represent samples collected from dogs in mock-vaccinated dogs at 24 and 48 hpi, respectively; lanes 3 and 4 represent samples collected from dogs immunized with LBNSE-dGM-CSF at 24 and 48 hpi, respectively; lanes 5and 6 represent samples collected from two dogs immunized with LBNSE-dGM-CSF at 72 hpi; lane 7 represents samples collected from a dog immunized with LBNSE-dGM-CSF at 96 hpi; lane 8 represents the positive control using the total RNA extracted from LBNSE-dGM-CSF infected NA cells as the template. For **D.**, lane 1 represents the positive control; lanes 2 and 3 represent the reagent controls of first round PCR and second round PCR for vRNA amplification, respectively; lane M represents DNA ladder marker; lanes 4 and 5 are the reagent controls of first round PCR and second round PCR for cRNA amplification, respectively. The tonsil was also used for detection of viral antigens by IHC using anti-rabies virus P antibodies and representative IHC results for mock (left) and LBNSE-dGM-CSF (right) groups are shown in **E.**, and the brown dots (pointed by black arrows) are positive for viral antigen detection.

No adverse signs were observed in dogs after vaccination with either the parent virus LBNSE or the recombinant LBNSE-dGM-CSF. To investigate if and where the recombinant LBNSE-dGM-CSF can replicate in the oral cavity, the tonsils, buccal mucosa and tongues were collected and viral RNA detected by nested RT-PCR at different time points post vaccination. As shown in Figure 2A, vRNA and cRNA were detected in the tonsils at almost all time points. No viral RNA was detected in the tongues or buccal mucosa from these animals except the detection of genomic RNA in the tongues at 48 hr after vaccination (Figures 2B and 2C), and Figure 2D is the internal reagent controls for the nested RT-PCR. Furthermore, IHC confirmed the result that viral antigen was detected in all the tonsil samples from dogs vaccinated with LBNSE-dGM-CSF (Figure 2E). All the above results suggest that the recombinant LBNSE-dGM-CSF replicates mainly in the tonsils where the virus most likely initiates the immune responses.

### Recruitment and activation of DCs and B cells in the peripheral blood after oral immunization

**Figure 3:**
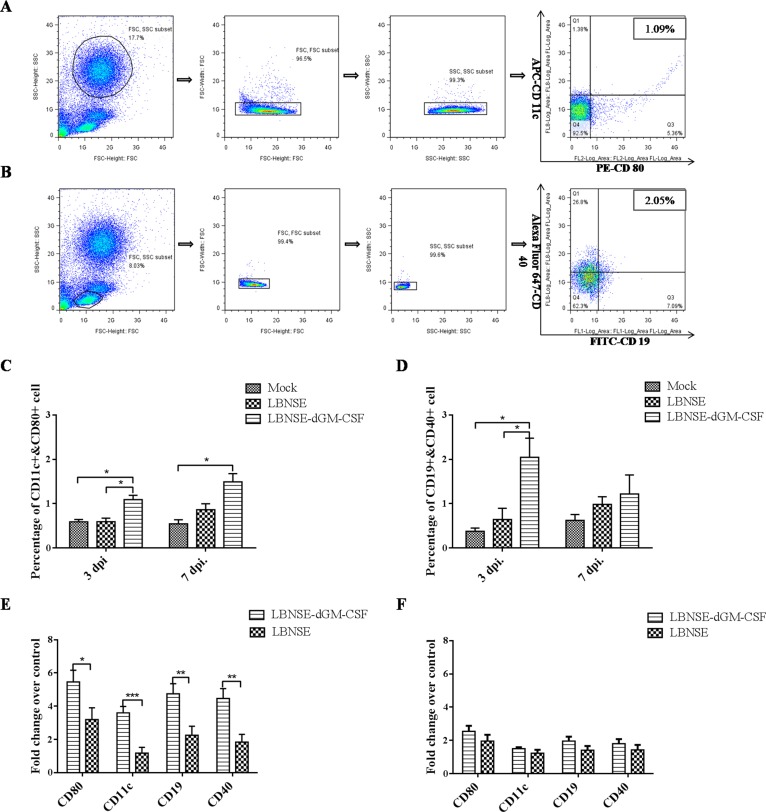
Measurement of DC and B cell activation in the peripheral blood after oral vaccination by flow cytometry and qRT-PCR Dogs were sham-immunized or orally immunized with 1×10^8^ FFU of LBNSE-dGM-CSF or LBNSE. Peripheral blood was collected on 3 and 7dpi. For flow cytometry, single cell suspensions were prepared and stained with antibodies for DCs (CD11c and CD80) or B cells (CD19 and CD40), and the representative gating strategy of DCs **A.** and B cells **B.** were displayed. The detailed analysis for activation of DCs **C.** and B cells **D.** were performed and presented. For qRT-PCR, PBMCs were isolated and total RNA was extracted, the mRNA level of surface co-stimulating markers of DCs (CD11c and CD80) and B cells (CD19 and CD40) at 3dpi **E.** and 7 dpi **F.** were detected using primers listed in S1 Table. Asterisks indicate significant differences analyzed by one-way ANOVA **C.** and **D.** or student T test **E.** and **F.** between the different groups. (*, *P* < 0.05; **, *P* < 0.01; ***, *P* < 0.001).

To investigate if expression of dog GM-CSF by RABV can recruit and activate more DCs and B cells than the parent virus after oral vaccination, peripheral blood samples from all the dogs were collected at 3 and 7 dpi and analyzed by flow cytometry. The representative gating strategies for detection of DCs and B cells are as shown in Figure 3A and 3B, respectively. As shown in Figure 3C and 3D, significantly more activated DCs and B cells were detected in the peripheral blood from dogs vaccinated with LBNSE-dGM-CSF than those from dogs vaccinated from LBNSE or from sham-immunized dogs at 3 dpi, while only significantly more DCs were detected in peripheral blood from dogs vaccinated with LBNSE-dGM-CSF than those from sham-immunized dogs at 7 dpi. Meanwhile, qRT-PCR was also performed to determine the mRNA level of surface co-stimulating molecules on DCs or B cells. As expected, the mRNA level of the markers for DCs (CD 11c and CD80) and B cells (CD19 and CD40) in the peripheral blood from dogs vaccinated with LBNSE-dGM-CSF are significantly higher than those from dogs vaccinated from LBNSE at 3 dpi, while no significant difference is detected at 7 dpi although the mRNA level of each marker detected in LBNSE-dGM-CSF group is still higher than that in the dogs immunized with the parent virus (Figure 3E and 3F). All these data indicate that the LBNSE-dGM-CSF can recruit and activate more DCs and B cells followed by the circulation of these cells in the peripheral blood than the parent virus LBNSE after oral vaccination, which is consistent with our previous studies in mice [38, 39].

### VNA induction and protection after oral vaccination

**Figure 4:**
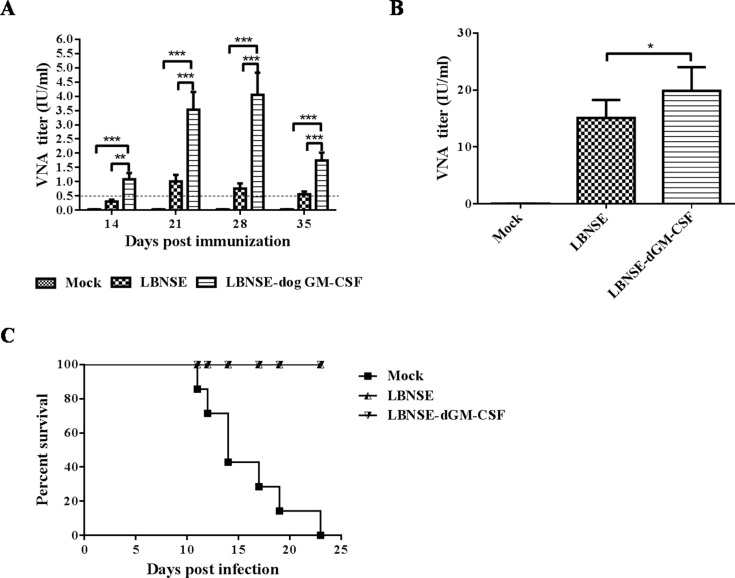
Detection of VNA titer after oral immunization and survivorship after challenge Dogs were orally immunized with 1×10^8^ FFU of LBNSE-dGM-CSF, LBNSE, or DMEM. Peripheral blood was collected at 2, 3, 4 and 5 weeks post immunization for VNA test by RFFIT **A.** At 5 weeks after immunization, immunized dogs were challenged with 4500 MICLD_50_ of wild-type RABV SXTYD01 by injecting it at masseter and temporal muscles. Peripheral blood was collected at 7 days post challenge and the serum was for VNA test **B.** After challenge, dogs were observed twice a day for 3 months and the numbers of survivors were recorded **C.** Asterisks indicate significant differences analyzed by one-way ANOVA between the different groups. (*, *P* < 0.05; **, *P* < 0.01; ***, *P* < 0.001).

To investigate if oral vaccination with LBNSE-dGM-CSF can induce higher levels of VNA than the parent virus, two groups of beagles were orally immunized with 10^8^ FFU of LBNSE-dGM-CSF or LBNSE and blood samples were collected at different time points after vaccination for the measurement of VNA. As shown in Figure 4A, significantly higher VNA titers were detected in dogs immunized with LBNSE-dGM-CSF than in those immunized with LBNSE at all the time points tested (p values are 0.0019, 0.0008, 0.0004, and 0.0005 at 14, 21, 28, and 35 dpi., respectively). Of note is that VNA titers in all dogs immunized with LBNSE-dGM-CSF were higher than 0.5 IU/ml (1.09±0.53 IU/ml) as early as 2 weeks post immunization. By contrast, only two of the six dogs immunized with LBNSE induced VNA titers equal to 0.5 IU/ml (0.30±0.16 IU/ml) (Table 1). The highest level of VNA (4.05±1.91 IU/ml) was detected at 28 dpi in dogs immunized with LBNSE-dGM-CSF, while the highest level of VNA (1.01±0.57 IU/ml) was detected at 21dpi in dogs immunized with LBNSE. All these animals were challenged with wild-type RABV SXTYD01. All the dogs in the mock-vaccinated group succumbed to rabies (RABV was detected in all brain samples of these dogs by direct fluorescence assay, data not shown), whereas, all the vaccinated dogs, either immunized with LBNSE-dGM-CSF or LBNSE, were protected from the lethal challenge (Figure 4C). The VNA level at 7 days post challenge was determined (Figure 4B). Significantly higher (*p* = 0.0381) VNA titers were detected in dogs immunized with LBNSE-dGM-CSF (geometric mean titer is 19.47 IU/ml) than in dogs immunized with LBNSE (geometric mean titer is 14.79 IU/ml). Taken together, the rRABV expressing dog GM-CSF can induce higher level of VNA than the parent virus in dogs after oral immunization.

**Table 1:** Summary of VNA titer and survivorship in each group

Groups	Dog ID	VNA^a^ titer (IU/ml)			
		14dpi	21dpi	28dpi	35dpi
LBNSE	1	0.50	1.14	0.87	0.66
	2	0.29	0.87	0.66	0.66
	3	0.13	0.29	0.17	0.17
	4	0.50	1.97	1.50	0.87
	5	0.17	0.66	0.50	0.50
	6	0.22	1.14	0.87	0.50
LBNSE-dGM-CSF	1	0.87	3.42	4.50	1.50
	2	0.66	3.42	5.92	2.60
	3	0.87	1.97	1.97	1.50
	4	1.50	5.92	5.92	2.60
	5	1.97	4.50	4.50	1.14
	6	0.66	1.97	1.50	1.14
Mock	1	0.02	0.03	0.02	0.02
	2	0.03	0.03	0.03	0.03
	3	0.02	0.02	0.03	0.02
	4	0.02	0.02	0.02	0.02
	5	0.03	0.02	0.03	0.03
	6	0.02	0.03	0.02	0.02
	7	0.02	0.03	0.02	0.03

a Virus neutralizing antibodies

## DISCUSSION

Our previous studies demonstrate that recombinant RABV expressing murine GM-CSF can induce significantly higher VNA titer than the parent virus after oral immunization in a mouse model [38]. In the present study, we constructed recombinant RABV expressing dog GM-CSF and found that this recombinant virus can induce robust immune responses and provide protection against challenge in dogs after oral immunization.

GM-CSF is a multi-functional immune modulator, playing an important role in the differentiation of the progenitor cells into DCs *in vitro* and *in vivo* [44] and it has been employed as an adjuvant to enhance the immunogenicity in many viral vaccines [39, 45-52]. In our studies, it was found that expression of GM-CSF by RABV could result in the recruitment and activation of more DCs than the parent virus in the murine [38, 39] as well as in the canine model via parenteral as well as oral routes. DCs are the most efficient antigen presenting cells, linking the innate and adaptive immune systems [53]. Activated DCs can present antigens to CD4^+^ T cells through MHC II, which subsequently stimulate B cells to generate antigen-specific antibodies [54]. Indeed, significantly more activated B cells and significantly higher levels of rabies VNA were detected in dogs vaccinated with LBNSE-dGM-CSF than in those vaccinated with the parent virus.

The observation that LBNSE-dGM-CSF stimulated higher VNA responses in orally immunized dogs than the parent virus LBNSE is very important. LBNSE is essentially SAG-2 that has been reported to be a poor VNA stimulator [26, 30, 31]. A VNA titer≥0.5 IU/ml has been regarded as a protection threshold for inactivated rabies vaccines [55]. For oral rabies vaccination with live attenuated rabies virus, some of the immunized animals with VNA titers below 0.5 IU/ml (or even undetectable) still could survive the lethal street RABV challenge as reported previously [26, 30, 31]. In another words, if the animals vaccinated orally with live attenuated RABV induce VNA titers≥0.5 IU/ml, they will be well protected from a lethal RABV challenge. Although oral immunization with the parent virus LBNSE protected all the dogs against challenge in this present study as it has been reported previously [26, 30, 31], only 2 out of the 6 dogs immunized with LBNSE developed VNA titer≥0.5 IU/ml by day 14 after immunization, while all the dogs immunized with LBNSE-dGM-CSF developed VNA titer≥0.5 IU/ml by this time. The 100% rate of sera-conversion after oral immunization with LBNSE-dGM-CSF makes it a superior oral vaccine over other oral vaccines in use or in development, particularly in monitoring vaccination efficacy. Upon challenge, the anamnestic response (VNA level) at 7 days post challenge in dogs immunized with LBNSE-dGM-CSF was still significantly higher than in those immunized with LBNSE.

Previous studies with VR-G have indicated that the oral cavity, particularly the tonsils, is the site for virus replication and thus initiation of immune responses after oral immunization [19, 56]. To examine if LBNSE-dGM-CSF is capable of replicating in the oral cavity, tonsils, tongues and buccal mucosa were collected at different time points after oral immunization and viral RNA measured by nested RT-PCR and IHC. It was found that the tonsils are the primary site for LBNSE-dGM-CSF replication, as has been observed in previous studies with SAG-2 [26]. Intriguingly, the level of activated DCs at 7 dpi is higher than that at 3 dpi (Figure 3C), which could attribute to the replication of vaccine viruses in the tonsil, allowing the continuous activation of DCs. The tonsil is an important secondary lymphoid organ that contains many cell types such as lymphocytes, macrophages and follicular DCs, which can trap immune complexes on the surface and facilitate B cell activation and maturation [57].

In summary, the recombinant LBNSE-dGM-CSF can replicate in the oral cavity, particularly in the tonsils. It is capable of inducing a robust VNA response by recruiting and activating DCs and B cells after oral vaccination and protecting immunized dogs from a lethal virus challenge, indicating that it has the potential to be developed as a safe and efficient oral rabies vaccine for dogs.

## MATERIALS AND METHODS

### Viruses, cell lines, antibodies and animals

A recombinant RABV (LBNSE), derived from attenuated SAD-B19 strain with two mutations at amino acid 194 and 333 of the G, was constructed as described previously [38, 39]. The wild-type virus SXTYD01 was isolated from a rabid dog in Shanxi province in China (a gift from Dr. Changchun Tu, Chinese Academy of Agricultural Sciences, Changchun, China) [58]. BSR cells, a cloned cell line derived from BHK-21 cells, were cultured in Dulbecco's modified Eagle's medium (DMEM) (Mediatech, Herndon, VA) supplemented with 10% fetal bovine serum (FBS) (Gibco, Grand Island, NY). Mouse neuroblastoma (NA) cells were cultured in RPMI 1640 medium (Mediatech) supplemented with 10% FBS. Fluorescein isothiocyanate (FITC)-conjugated anti-RABV N protein antibodies were purchased from Fujirebio Diagnostics, Inc. (Malvern, PA). APC-anti-CD 11c (clone BU15) was purchased from eBioscience (San Diego, CA), PE-anti-CD80 (clone 16-10A1) from BD Pharmingen (San Jose, CA), FITC-anti-CD19 (clone MB19-1) from Abcam (Shanghai, China), and Alexa Fluor 647-anti-CD40 (clone LOB7/6) from AbD (North Carolina, USA). Eight-month-old female purpose-bred beagles (not rabies vaccinated) were purchased from Hubei Center for Disease Control (CDC), Wuhan, China and individually housed according to the protocols approved by the Institutional Animal Care and Use Committee of Hubei Province (permit number: D2014003).

### Construction of recombinant RABV expressing dog GM-CSF

The recombinant RABV expressing dog GM-CSF, designated as LBNSE-dGM-CSF, was constructed as described in previous studies [38, 39]. Briefly, dog GM-CSF gene was cloned and then inserted into the genome of the parent virus vector pLBNSE, derived from SAD-B19 with the deletion of the long non-coding region between RABV G and L genes, using BsiWI and NheI sites added in pLBNSE. The full length infectious clone of LBNSE-dGM-CSF was transfected into BSR cells along with four helper plasmids (expressing N, P, G, L of the parent virus LBNSE, respectively) using the SuperFect transfection reagent (Qiagen, Valencia, CA) following the procedures described in previous studies [39, 40].

### Vaccination and challenge

Eight-month-old, non-rabies vaccinated, healthy beagles were randomly divided into three groups, with seven dogs in the mock group and six dogs in each vaccinated group that were either immunized with LBNSE or LBNSE-dGM-CSF. Vaccination was carried out when the maternal VNA level for RABV declined below 0.5 IU/ml in all dogs. In vaccinated groups, dogs were either orally immunized with 10^8^ FFU of LBNSE or LBNSE-dGM-CSF using a needle-free syringe. Dogs in the mock group were given DMEM orally. Peripheral blood samples were collected at different days post immunization (dpi) for detection of VNA level and activation of DCs and B cells. All the dogs were challenged with 4500 MICLD_50_ (50% mouse intracerebral lethal dose) of wild-type RABV SXTYD01 by injecting the virus at the masseter and temporal muscles at 35 dpi, and were observed twice daily for three months. The dogs were sedated and euthanized when any rabies clinical signs, such as ataxia, paresis, and paralysis were observed, and survivorship was recorded and analyzed.

### Virus titration

The RABV was titrated in NA cells using a direct fluorescent antibody assay. Briefly, NA cells seeded in 96-well plates were inoculated with serial 10-fold dilutions of virus and incubated for 48 h at 34°C. After incubation, cells were fixed with 80% ice-cold acetone and then stained with FITC-labeled rabies virus N protein-specific antibodies. Antigen-positive foci were determined under a fluorescence microscope, and virus titer was calculated as focus-forming units/mL (FFU/mL). All the titrations were carried out in quadruplicate.

### Rapid fluorescent focus inhibition test (RFFIT)

RFFIT was performed to determine the VNA level in the peripheral blood as described previously [38]. Briefly, 50 μl of serial five-fold dilutions of serum were prepared in Lab-Tek Chamber slides (Nalge Nunc International, Rochester, NY). Fifty FFD_50_ (50% Fluorescing Foci dose) of challenge virus standard (CVS-11) was added to each chamber and incubated at 37°C for 90 min. After incubation, 10^5^ of NA cells were added into each chamber and the slides were incubated at 37°C for 24 hrs. Then the culture medium in each chamber was discarded and the cells were fixed with ice-cold 80% acetone at −20°C for 15 min. After washing with PBS for three times, the cells were stained with FITC-conjugated anti-RABV N antibodies for 1 hr at 37°C. Twenty fields in each chamber were observed under a fluorescent microscope, and the 50% endpoint titers were calculated according to the Reed-Meunch formula [59]. The values were compared with that of a reference serum (obtained from the National Institute for Biological Standards and Control, Herts, UK) and normalized to international units (IU/ml).

### Flow cytometry

To investigate if DCs and B cells were activated after oral immunization, peripheral bloods were collected and the red blood cells lysed by Red blood cell Lysing Buffer (Beyotime, China). Single-cell suspensions were prepared at10^6^ cells/ml in Stain Buffer (BD Pharmingen) and then stained with antibodies against CD40, CD19, CD11c, and CD80 at 4°C for 30 min. After staining with antibodies, cells were washed three times and then fixed with 1% paraformaldehyde. Flow cytometery was performed on LSR-II flow cytometer (BD Bioscience) and data analyzed by BD FACSDiva (BD Pharmingen) and FlowJo software (Tree Star).

### Quantitative real-time PCR (qRT-PCR)

To quantify the mRNA levels of surface co-stimulating molecules on DC or B cells in the peripheral blood after oral vaccination, qRT-PCR was performed in ABI Prism 7500 fast sequence detector system with Power SYBR green PCR master mix (Applied Biosystems). Blood samples were harvested at different time points and total RNA extracted from the isolated peripheral blood mononuclear cells (PBMCs). The reverse transcriptase and DNA polymerase were utilized from a one-step Brilliant II SYBR green qRT-PCR master mix kit (Stratagene). Each reaction was carried out in duplicate with approximately 100 ng of RNA and 5 nM each primer pairs as shown in S1 Table. Amplification was carried out at 50°C for 2 min and 95°C for 10 min, followed by 40 cycles in two steps: 95°C for 15 s and 60°C for 1 min. The mRNA copy numbers of each surface co-stimulating molecule were normalized to those of the housekeeping gene Δ-actin. Expression Levels of each molecule in each vaccinated group (LBNSE or LBNSE-dGM-CSF) were presented as the fold change over that detected in the blood samples from mock-immunized group.

### Nested reverse transcription (RT) PCR

To investigate if viral replication occurred in the oral cavity after oral immunization, nested RT-PCR was performed using the primers as described in previous studies [26] (listed in S2 Table). Immunized dogs were euthanized at different dpi, and tonsils, tongues, and buccal mucosa were collected for the detection of genomic RNA (vRNA) and sense transcribed RNA (cRNA). Total RNA were extracted from each tissue sample and the reverse transcription was performed using primers 509 and 304 for vRNA and cRNA detection, respectively. The primers 509 and 304 and nested primers 504 and 105 were employed to amplify RABV N gene of rabies. The PCR products were analyzed by agarose gel electrophoresis.

### Immunohistochemistry (IHC)

To further confirm if viral replicates in the tonsils, immunohistochemistry was carried out. Briefly, tonsil samples were fixed in 10% neutral buffered formalin and then paraffin embedded for coronal sections. De-paraffinization was performed by heating the slides at 60°C for 25 min and then placing the slides in CitriSolv (Fisher Scientific, PA) for 5 min, three times, followed by drying the slides. After de-paraffinization, the slides were heated above 90°C for 20 min in antigen unmasking solution (Vector Laboratories, CA) and cooled down to room temperature. Anti-RABV P monoclonal antibody was used to detect RABV antigen. Biotinylated secondary antibodies were then reacted with the primary antibodies. To localize the biotinylated antibody, the avidin-biotin-peroxidasecomplex (Vector Laboratories, CA) was employed. Finally, color development was carried out using diaminobenzidine (DAB) as a substrate.

### Ethics statement

The animal experiments were carried out in strict accordance with the protocols (permit number: D2014003) approved by the Institutional Animal Care and Use Committee of Hubei Province. The animal care and maintenance were in compliance with the recommendations in the Regulations for the Administration of Affairs Concerning Experimental Animals of P.R. China.

### Statistical analysis

Statistical significance among different experimental groups was analyzed by one-way ANOVA or student T test using GraphPad prism software.

## SUPPLEMENTARY MATERIAL TABLES


