# Supplementary material for: Recombinant rabies virus expressing dog GM-CSF is an efficacious oral rabies vaccine for dogs
Source: Oncotarget. 2015 Sep 30;6(36):38504–16. doi: 10.18632/oncotarget.5904 (PMC4770717; doi:10.18632/oncotarget.5904)
Supplement: Supplementary file 1 [file oncotarget-06-38504-s001.pdf]

Recombinant rabies virus expressing dog GM-CSF is an efficacious oral rabies vaccine for dogs

Supplementary Material

S1Table: Primers used for detection of surface co-stimulating molecules on DC (CD11c, CD80) and B cell (CD19, CD40) by qRT-PCR

**Name**	**Upper primer(5'–3')**	**Lower primer(5'–3')**
β-actin	CTGAAGTACCCCATTGAGCACGGCA	GGGTCATCTTCTCACGGTTGGCCTT
CD19	CCGTCGAGATGCAGCTGAAGGTCAT	GTCTGGTGGGATCGGTCATTCGCTT
CD11c	CCATCTACACCGTGATCAGCAGCCA	ACAGGCATCCCATTCAGCTCCACAG
CD40	TCTACCCAGAACCACGCACTGCATG	CTGACGTGCCCTCCTTCTCGACATG
CD80	GTTTCCCAAGACCCGGACACTGAGT	GGGAGGGTGTGGCTCGACTGATTTT

S2Table: Primers used in nested RT-PCR for detection of virus genomic RNA and sense transcribed RNA

**Name**	**Primer (5’–3’)**
509	GAGAAAGAACTTCAAGA
304	TTGACGAAGATCTTGCTCAT
504	TATACTCGAATCATGATGAATGGAGGTCGACT
105	TTCTTATGAGTCACTCGAATATGTCTTGTTTAG
